# Mouse Models of Muscle Fibrosis: Mechanisms, Methods, and Applications

**DOI:** 10.3390/biomedicines14020328

**Published:** 2026-01-31

**Authors:** Sarah E. DiIorio, Mia J. Fowler, Bill Young, Michelle F. Griffin, Michael T. Longaker

**Affiliations:** 1 Hagey Laboratory for Pediatric Regenerative Medicine, Division of Plastic and Reconstructive Surgery, Department of Surgery, Stanford University School of Medicine, Stanford, CA 94305, USA; diiorios@stanford.edu (S.E.D.); fowlerm@stanford.edu (M.J.F.); younbil@stanford.edu (B.Y.); 2 Institute for Stem Cell Biology and Regenerative Medicine, Stanford University School of Medicine, Stanford, CA 94305, USA

**Keywords:** skeletal muscle, muscle fibrosis, mouse model, volumetric muscle loss, laceration, myotoxin

## Abstract

Skeletal muscle injuries are common and some are able to regenerate due to satellite cells, the muscle stem cell population. However, in cases of severe muscle injury, complete tears, or muscle loss via trauma, muscles can undergo fibrosis and long-term compromise of their structure and function. The development of animal models has been key to understanding the pathways involved in muscle injury, fibrosis, and repair. In this review, we discuss the animal models currently used, with a focus on those most applicable to studying muscle fibrosis after traumatic injury. We summarize the approach, findings, and limitations of the most widely used models, including volumetric muscle loss, laceration, and myotoxin injection studies, and provide a brief description of ischemia/reperfusion, crush injury, freeze injury, and dystrophy models. We summarize the histological, cellular, molecular, and functional outcome measures commonly used in the field and outline areas for translation and future work. An expansion of current studies to specifically focus on muscle fibrosis will surely elucidate novel mechanisms for reducing debilitating fibrosis and promoting regeneration.

## 1. Introduction

**Figure 1:**
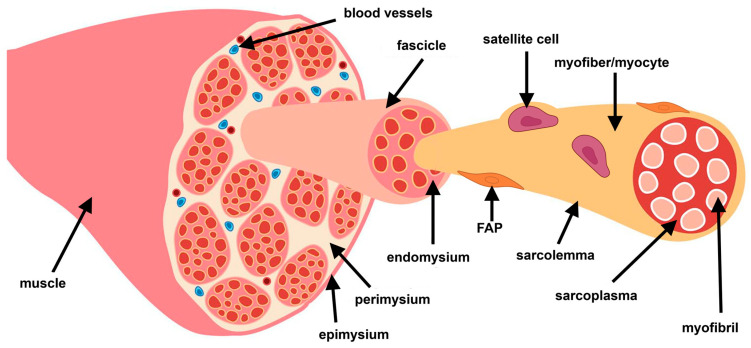
Diagram of organizational structure of muscle. Muscle fibers are made up of multiple fascicles, fascicles are made up of multiple myofibers, and myofibers contain multiple myofibrils. ECM layers include the epimysium, perimysium, and endomysium. Fibroadipogenic progenitors (FAPs) reside between myofibers, and satellite cells reside between the sarcolemma and basement membrane. Adapted from [3]. Created in BioRender. Diiorio, S. (2026) https://BioRender.com/cdarg2a.

Skeletal muscle is a dynamic tissue that is critical to the structure, mobility, and metabolism of the body [1]. Approximately 40% of human body weight is skeletal muscle, and muscles contain 50–75% of all body proteins [1]. The key functions of skeletal muscle are largely made possible by the anatomical structure and organization of muscle tissue. Specifically, skeletal muscle has several levels of organization: first is the muscle itself, which is made of multiple fiber bundles called fascicles (Figure 1). Fascicles are made up of individual muscle fibers, also called myocytes or myofibers. These myofibers are each surrounded by a cell membrane called the sarcolemma and contain the basic myofilament structures that allow muscles to contract [1,2]. In addition to the myofibers, there is also an extracellular matrix (ECM) that supports muscle structure and function. ECM layers surround each layer of muscle structure, with the epimysium surrounding the muscle, perimysium surrounding each fascicle, and endomysium surrounding each myofiber [1,2,3].

Supporting cells in the muscle include endothelial and immune cells, as well as quiescent muscle stem cells (MuSCs), known as satellite cells [4]. These cells reside between the sarcolemma of myofibers and the basal lamina of the basement membrane and respond rapidly to injury by differentiating into myocytes (Figure 1) [4,5]. Other important cells are the fibroadipogenic progenitors (FAPs), which reside in the interstitial space between myofibers and can differentiate primarily into fibroblasts or adipocytes (Figure 1) [6]. These cells are central to the maintenance of muscle ECM and respond to signals such as transforming growth factor-β1 (TGF-β1) to produce new ECM after injury [7].

**Figure 2:**
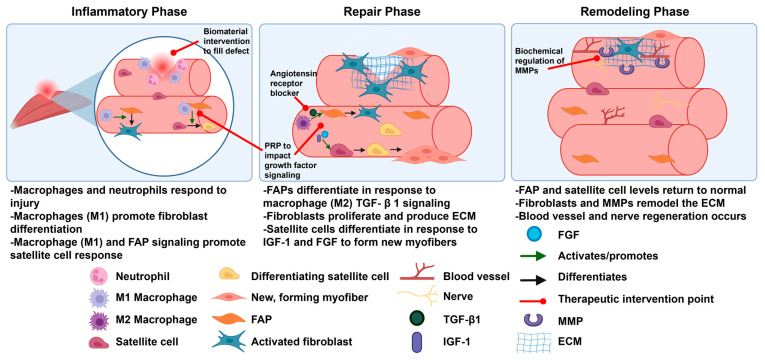
Schematic of the phases of healing and cells, pathways, and signaling molecules involved. Targets of therapeutic interventions are also highlighted. Abbreviations: FAP, fibroadipogenic progenitor; TGF-β1, transforming growth factor-β1; IGF-1, insulin-like growth factor-1; FGF, fibroblast growth factor; MMP, matrix metalloprotease; ECM, extracellular matrix. Created in BioRender. Diiorio, S. (2026) https://BioRender.com/pyy0q50.

Skeletal muscles undergo damage through several mechanisms including sports injuries such as contusion or strain and trauma such as volumetric muscle loss or laceration [1,8,9]. Muscle injuries are common, accounting for 40.9% of all recorded injuries at international sports championships between 2007 and 2015 [10]. Muscle trauma is also common, in particular, among military veterans: Corona et al. reported that muscle conditions accounted for 65% of all orthopedic injuries, and volumetric muscle loss trauma accounted for 92% of muscle injuries in a cohort of military veterans [11]. Similarly to other tissues [12,13], muscles heal through a three-phase process: first, the inflammatory phase, next, the repair or regeneration phase, and finally, the remodeling phase (Figure 2) [14]. In the inflammatory phase, muscle injury leads to myofiber necrosis, hematoma formation, and immune cell infiltration [2,14]. Macrophages first contribute to inflammatory signaling as M1 macrophages, which secrete Thy1 cytokines and other inflammatory markers [14]. Later, anti-inflammatory M2 macrophages secrete TGF-β1, signaling the start of the repair phase [14,15]. In this phase, FAPs proliferate and differentiate into activated fibroblasts in response to the secreted TGF-β1 [2]. Fibroblasts then contribute to the formation of temporary ECM components such as fibrin, fibronectin, collagen type I, collagen type III, and proteoglycans [2]. New myofibers begin to form as satellite cells differentiate in response to insulin-like growth factor-1 (IGF-1) and fibroblast growth factors (FGFs) [14]. Finally, in the remodeling phase, matrix metalloproteases (MMPs) degrade the ECM, which promotes inflammation and the activation of satellite cells to repair myofibers [2]. Vasculature and nerve connections are restored, and remodeling of the ECM allows for strengthening of both the muscle and normal levels of ECM that remain [2,14]. Each of these phases provides points for therapeutic intervention to reduce fibrosis (Figure 2) [16,17,18].

Unlike other tissues that always heal with a scar, muscles are highly regenerative after injury due to the previously mentioned satellite cells [4]. However, chronic or extreme injury such as surgery, car accidents, or battlefield injuries can lead to formation of scars in the muscle, also called fibrosis [6]. Fibrosis is defined in muscle by an overproduction and deposition of ECM, in particular of collagen proteins in the endomysium and perimysium [19,20]. While all muscle has healthy ECM holding together the myofibers, blood vessels, and nerves, an overproduction of these matrix proteins can lead to impaired muscle function and increased risk of re-injury [2,21]. This can be extremely debilitating to patients, preventing them from playing competitive sports, completing work tasks, and even performing day-to-day activities. Muscle fibrosis is largely thought to be caused by a disruption in the normal function of FAPs [6,22]. Thus, much work is being performed to understand the mechanisms that influence the FAP response.

To better understand the mechanisms of muscle injury, fibrosis, and repair, multiple translational animal models have been developed. Each of these models answer different questions surrounding muscle trauma, degeneration, fibrosis, or regeneration. While pigs, canines, sheep, rats, zebrafish, and mice have been used for in vivo animal models, mice are among the most used due to their ease of breeding, low cost, mammalian physiology, and the availability of established genetic manipulation approaches [2,23]. In this review, we will primarily focus our discussion on mouse models of skeletal muscle injury and provide an understanding of each model’s current uses, limitations, outcome measures, and clinical applicability. We will have a specific focus on models’ ability to study skeletal muscle fibrosis and repair. We identified the literature for this review broadly using multiple search terms in PubMed with a focus on publication in the last ten years. The list of individual search terms for each model type is detailed in Appendix A.

## 2. Mouse Models of Muscle Injury

Herein, we summarize the most common mouse models of muscle injury. Specific information about mouse strains was not included, as surgical procedures remain unchanged regardless of strain. Studies use varying strains depending on the pathway of interest. Table 1 summarizes the main takeaways for the volumetric muscle loss, laceration, and myotoxin injection models.

### 2.1. Volumetric Muscle Loss

Volumetric muscle loss (VML) is defined as a significant loss of muscle tissue that cannot be regenerated fully and results in functional impairment [52]. Commonly seen following traumatic accidents, oncologic resections, or degenerative musculoskeletal diseases [29,53], volumetric muscle loss (VML) has been increasingly used to investigate muscle fibrosis. 

Throughout the literature, various methods have been utilized to create a standardized excision of muscle, including biopsy punch instruments [24,54,55] and wedge resections [30,56]. This generates either full-thickness [31,57] or partial-thickness muscle defects [30]; however, there is significant variation among studies (Table 1). While VML models commonly use defect diameters ranging from 1 to 5 mm [25,58], larger defects may be created using multiple adjoining punches to increase the total injury area [59]. Additionally, most studies involve the ablation of 20–30% of an individual muscle’s mass [30,34,60], with Anderson et al. demonstrating that roughly 15% of a muscle’s mass must be removed as the critical size for fibrosis to occur [61]. This closely mirrors the 20% of a muscle’s mass that must be lost to cause human muscle fibrosis [62]. The biologic response to a VML is characterized by an absence of reinnervation, insufficient defect regeneration, and nonhealing histomorphology, such as collagen fibrosis and fatty infiltrate [29]. This non-regenerating, chronic defect is accompanied by long-term deficits in muscle mass and force production, and can also result in broader physiologic changes such as shifts toward increased slow-twitch fiber composition and impaired whole-body metabolism [57]. Experimental VML injuries have commonly been studied in the mouse quadriceps and tibialis anterior (TA), likely due to their superficial anatomy, enabling easy access for quantitative measurements of the defect and tissue harvest [59,61].

Characterizing the fibrotic response following a VML injury is critical for guiding potential therapeutic interventions. Prior studies have demonstrated distinct temporal patterns in wound healing after VML, with angiogenic responses preceding myogenic processes [26]. Time-series transcriptomic analyses reveal persistent changes after VML, with sustained upregulation of pro-inflammatory and ECM remodeling pathways, and suppression of genes associated with metabolic pathways [63]. Spatial sequencing of muscle fibrosis after a VML has further demonstrated upregulation of collagen deposition genes within the defect region, with macrophages identified as key mediators of pro-fibrotic signaling [64]. Genomic profiling of key wound healing genes following VML has shown similar genetic profiles between VML and uninjured muscles, highlighting a lack of regenerative processes occurring after injury [29]. Collectively, these findings highlight a dysregulated healing response characterized by persistent inflammation, excessive fibrosis, and limited myofiber regeneration.

**Figure 3:**
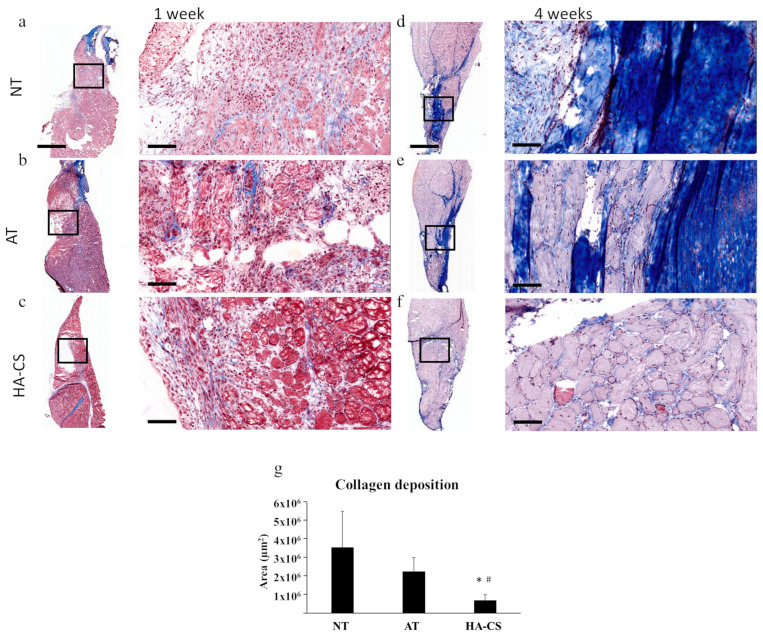
Volumetric muscle loss model is a good representation of fibrosis. (**a**) Masson’s trichrome staining from VML model showing collagen deposition at (**a**–**c**) 1 week and (**d**–**f**) 4 weeks after injury. (**a**,**d**) are from the no treatment group, (**b**,**e**) are from the autograft treatment group where the biopsied muscle was sutured back into the defect, and (**c**,**f**) are from the hydrogel group, where a HA-CS hydrogel was sutured into the defect. (**g**) Quantification of collagen deposition shows that fibrosis was significantly reduced at 4 weeks post surgery, when treated with HA-CS hydrogel. Overall, the NT and AT groups show more fibrosis after four weeks than HA-CS. Scale bar: 2 mm for full-scan sections (left of **a**–**f**) and 100 μm for magnified images (right of **a**–**f**). * *p* < 0.05 compared to NT group; # *p* < 0.05 compared to AT group. Abbreviations: VML, volumetric muscle loss; NT, no treatment; AT, autograft treatment; HA-CS, hyaluronic acid–chondroitin sulfate. Data from [55].

Several studies have explored therapeutic approaches aimed at modulating the inflammatory and fibrotic responses after VML based on this research. Castor-Macias et al. found that repleting inflammatory regulators, such as Maresin-1, attenuated the inflammatory response and increased myogenic cell proliferation, driving reductions in wound fibrosis [24]. Additional investigations have examined antifibrotic agents such as nintedanib [65], inhibition of TGF-β signaling [64], and the use of cardiosphere-derived cells [27] as potential means of improving muscle regeneration and functional recovery. Biomaterial-based approaches have also shown promise; implantation of biologic ECM scaffolds has been reported to promote a constructive remodeling response after VML with improved myogenesis and angiogenesis [59,66,67,68]. Separately, hydrogel scaffolds have also been investigated as a therapeutic strategy, with studies reporting restoration of muscle fibers and, importantly, reductions in collagen deposition and fibrotic tissue formation (Figure 3) [55,69,70,71]. Finally, stem cell-based therapies have been explored using a variety of delivery strategies, including incorporation within biomaterial scaffolds and transplantation of minced muscle tissue. These studies have shown restoration of muscle mass and satellite cell populations with concomitant reductions in fibrosis [28,30,32,33]. The lack of treatments that can restore muscle function for patients in the clinic underscores the continued need for exploration in the space of biomaterials and cell therapies for VML. Though the current standard of care for patients in the clinic is microvascular free flaps to reconstruct the defect, this does not fully restore muscle strength and function [11]. VML defects therefore present an optimal use case for biomaterial intervention.

Compared to other models of skeletal muscle injury, the critical loss of muscle mass seen in VML is a defining feature, and results in a heightened fibrotic response [72]. This increased fibrosis is thought to be due to the disruption of the basal lamina, as well as the loss of growth factors and muscle stem cells from the microenvironment [62]. In contrast, chemical (e.g., myotoxin) or physical (e.g., laceration or freeze) models involve a shorter-lived inflammatory response following injury, which ultimately drives a more robust regenerative response [24,29,50,73].

However, the VML model is not without limitations; the standardized excision of tissue may not represent the complex, heterogeneous trauma commonly seen in real-world VML injuries [74]. The current literature highlights a need for further research to identify approaches for improved recovery after VML. Still, VML provides a reproducible mechanism of inducing muscle fibrosis that is fundamentally different from chemical or laceration models of muscle injury [31].

### 2.2. Laceration

While less common than VML models, laceration models have been used to study skeletal muscle injury. These models involve a transverse cut through muscle fibers without tissue removal, distinguishing them from VML. The surgical approach enables controlled study of direct mechanical deformation that closely mimics severe human muscle trauma and is highly reproducible [37,75]. Importantly, laceration has been shown to induce fibrotic scar formation, allowing investigation of how fibrosis limits regeneration and function, and study of cellular and molecular pathways governing the balance between fibrosis and regeneration. Small animal models of muscle laceration are most used, including both rat and mouse models, making this an accessible and low-cost approach. Surgical approaches most commonly involving a transverse incision through the gastrocnemius, with some variation in surgical technique. The first mouse laceration model described an incision at 60% of muscle length from the distal insertion, extending through 75% of the width and 50% of the thickness [35,36]. More commonly, studies employ a full-thickness transection across 100% of the muscle width [38,39,75,76], while one study reported a partial transection limited to the lateral portion of the muscle (Table 1) [41].

Early studies in laceration models helped guide clinical management [35], identified a potential role of antifibrotic agents in mitigating fibrosis [37], and helped characterize the molecular pathways involved in skeletal muscle fibrosis and regeneration [39,77,78]. This included characterizing the TGF-β pathway as a key fibrotic mediator driving fibrosis and fibrotic differentiation of muscle-derived cells [38,75]. More recently, a laceration model was used by Murray et al. to identify integrin regulation of *PDGFRβ*+ mesenchymal cells in skeletal muscle fibrosis, offering a targeted pathway for inhibiting fibrosis without global TGF-β blockage [40].

Despite consistent characterization of muscle injury, approaches to muscle closure are poorly described. The original murine model reported closure using a modified Kessler stitch with polydioxanone 7-0 sutures [35]. However, some studies mention superficial muscle closure without technical detail [36,79] and most omit closure methods entirely. This variability is important as, without surgical closure, tonic muscle forces may prevent re-approximation, creating a defect resembling VML. While loss of structural framework is a known driver of fibrosis and is well studied in VML models, laceration models uniquely capture clinical scenarios in which muscle is reapproximated yet still develops fibrosis and incomplete regeneration. Notably, two studies describing “laceration” models in fact used VML-type injuries [79,80], highlighting inconsistent terminology that may obscure interpretation and comparison across models.

Although laceration models offer pathophysiological relevance and reliably induce fibrosis, small animal models of skeletal muscle laceration often heal more completely than humans and fail to reproduce the severity of fibrosis seen clinically [36,76]. This limitation may explain the shift toward VML models, which produce more severe injury but may have less pathophysiological relevance to typical human muscle trauma [80].

### 2.3. Myotoxin Injection

Myotoxin injury models involve the injection of a myotoxic agent, namely snake venoms, local anesthetics, and chemical toxins, into the muscle. The two most common venom-derived toxins are cardiotoxin (CTX), a protein kinase-C inhibitor, and notexin (NTX), a phospholipase A_2_ [50,81]. Bupivacaine is the most commonly used local anesthetic, and barium chloride (BaCl_2_) is the most used chemical toxin [50,82]. While each of these myotoxins function through slightly varied mechanisms, they ultimately all lead to a disruption of calcium homeostasis in myofibers, sustained or dysregulated muscle contraction, and muscle damage [49,82,83]. The majority of myotoxin models are conducted in mice, with most using an injection into the tibialis anterior (TA) [42,43,46,50,51]; however the gastrocnemius [44] or extensor digitorum longus (EDL) [84] may also be used (Table 1).

**Figure 4:**
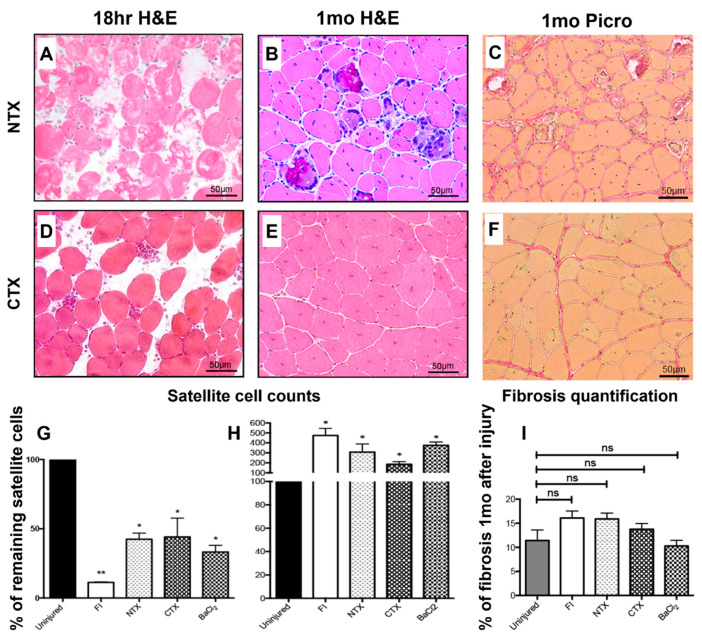
Myotoxin injection models and freeze models do not result in long-lasting fibrosis. (**A**,**B**) Representative H&E from (**A**) 18 h and (**B**) 1 month after NTX injection into the TA. (**C**) Representative picrosirius red imaging from 1 month after NTX injection showing minimal fibrosis. (**D**,**E**) Representative H&E from (**D**) 18 h and (**E**) 1 month after CTX injection into the TA. (**F**) Representative picrosirius red imaging from 1 month after CTX injection showing minimal fibrosis. (**G**) Satellite cell counts from 18 h and (**H**) satellite cell counts from 1 month after various myotoxin exposure. (**I**) Fibrosis quantification from 1 month after various myotoxin exposure. Levels were calculated from picrosirius red images. Significance: * *p* < 0.05, ** *p* < 0.01, ns—no significance. Abbreviations: NTX, notexin; CTX, cardiotoxin; FI, freeze injury; BaCl_2_, barium chloride; H&E, hematoxylin and eosin; Picro, picrosirius red; TA, tibialis anterior. Data adapted from [50].

Myotoxin injection mouse models are widely used due to their high reproducibility and utility in studying muscle regeneration [82]. The toxins act by inducing myofiber-specific damage, which spares the basal lamina and surrounding blood vessels and creates an environment to primarily study the myofiber and satellite cell response [82]. Myotoxin studies have shown the importance of pathways such as CREG1 [42], STAT3 [85], and MEGF10 [51] on satellite cell-mediated regeneration of muscle after injury. Other studies have used mytoxins to study related topics such as the role of macrophages and the immune system on muscle healing [46] and the impact of analgesics on muscle function and activity levels after injury [83]. Several studies have also been performed comparing various types of myotoxin responses to each other [47,50,82]. Interestingly, Hardy et al. showed that the varied mechanisms of action of different toxins led to varying responses in the satellite cell counts after injection (Figure 4). While they all had a significant drop in satellite cells within 18 h of injury, CTX-injected muscles had a similar satellite cell count to uninjured muscles at 1 month after injury, NTX-injected muscles had an elevated satellite count at 1 month, and BaCl_2_-injected muscles had the peak of their satellite cell count at 1 month (Figure 4G,H) [50]. By three months, CTX-injected muscles had steadily increased to double the satellite cell count compared to normal muscle, NTX-injected muscles had 2–3 times the satellite cell count than normal, and BaCl_2_-injected muscles exhibited a decrease compared to 1 month [50]. Broadly, histology looked relatively normal at 1 month, with granulomatous reaction to calcium present in NTX only (Figure 4A–F). However, morphometric analysis did show some loss of muscle fibers compared to normal in all groups at the 1- and 6-month timepoints [50].

Drawbacks of the myotoxin injection models include their decreased applicability toward clinical scenarios, as it is extremely rare to have venom exposure in humans. There are several other myotoxins that are more common exposures in humans, but many have broader systemic effects that make them less straightforward to study in mice [86]. In addition, from the perspective of studying muscle fibrosis, these models are not as useful compared to VML or other systems. This is because most myotoxin models create transient fibrosis and ultimately result in near total regeneration of the original muscle [2]. Even with repeated or increased dosage of myotoxins, skeletal muscle has been shown to fully regenerate [50]. Still, they provide a useful mechanism to understand the fundamentals of muscle regeneration and satellite cell behavior. Several studies have used myotoxin injection study FAPs [45,48]. Although this may at first appear surprising due to the lack of long-lasting fibrosis in these models, FAPs can still influence myogenesis through an increase in myogenic factors [48]. Myotoxin models therefore enable investigation of the early cellular injury response, including the role of FAPs.

### 2.4. Other Models

Several additional experimental approaches have been used to study skeletal muscle injury, fibrosis, and regeneration, including ischemia/reperfusion (I/R) and crush injury models. I/R models induce muscle damage through prolonged limb ischemia caused by sustained compression or vascular occlusion followed by restoration of perfusion [87]. Crush injury models typically use invasive or noninvasive physical compression to directly induce muscle damage [88]. Clinically, crush and I/R injuries are complex and are characterized by extensive myonecrosis in addition to fibrosis, often accompanied by systemic manifestations secondary to muscle breakdown [89]. Accordingly, most experimental studies using crush and I/R models have focused on inflammation and acute injury responses over short timeframes [87,90]. Although highly relevant for modeling severe traumatic injury, the profound local inflammation and systemic effects inherent to these models likely limit their suitability for isolating and studying muscle fibrosis as a primary outcome.

Another model is freeze injury, in which a probe is cooled using liquid nitrogen and placed on the mouse muscle [91]. The main advantage of this model is that it damages all cell types in the area of injury and is more focal than myotoxin injection [50]. Therefore, freeze injury can be leveraged to study cell migration in muscle after injury [50,91]. Interestingly, a previous rat study using freezing has shown that the muscle progenitor cells were not able to migrate from surrounding muscle after a complete ablation of the EDL unless a physical connection was made [92]. However, other models that use freeze ablation of only part of a muscle show that inflammatory, FAP, and satellite cells migrate into the injury [93]. Similarly to the myotoxin models, Hardy et al. showed only minor reduction in myofiber count and mild fibrosis at 1 month after freeze injury, and normal histology at 3 and 6 months, making it a less ideal system to study fibrosis [50].

A commonly used approach to study muscle fibrosis involves genetic models of dystrophic muscle disease, most notably Duchenne muscular dystrophy (DMD), a fatal X-linked recessive disorder characterized by chronic and progressive muscle fibrosis. The mdx mouse harbors a point mutation in exon 23 of the murine *Dmd* gene, resulting in the absence of functional dystrophin that closely recapitulates key aspects of the human disease phenotype. This leads to progressive muscle degeneration with replacement of functional myofibers by adipose and fibrotic tissue [94,95,96,97]. Methods have been proposed to combine genetic models with mechanical injury to further exacerbate fibrotic remodeling and more closely replicate DMD; however, these models are not commonly used [98,99]. However, because dystrophic models reflect impaired regeneration in a genetically diseased muscle rather than fibrosis arising in otherwise healthy tissue, they are less applicable for studying injury-specific fibrosis and were therefore not a focus of this review.

Age is also an important modifier of muscle regeneration and fibrosis, as aging is associated with impaired regenerative capacity and altered fibrotic responses. Some studies address this impact of aging on muscle atrophy and healing by conducting models in aged mice. However, because most injury and trauma-specific fibrosis studies in mouse muscle have been primarily conducted in young animals, age-related effects on muscle fibrosis were not addressed in this review. We also acknowledge that there may be other mouse models used to study muscle fibrosis that we did not discuss as they were beyond the scope of this review.

## 3. Outcome Measures

**Figure 5:**
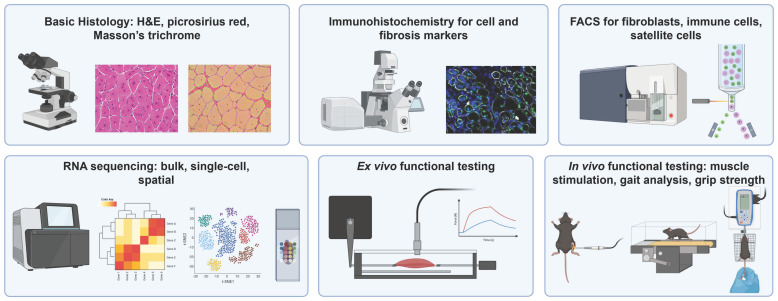
Schematic of outcome measures commonly used in muscle injury and fibrosis studies. Abbreviations: H&E, hematoxylin and eosin; FACS, fluorescence-activated cell sorting. H&E and picrosirius red data adapted from [50]. Immunohistochemistry data adapted from [24]. Created in BioRender. Diiorio, S. (2026) https://BioRender.com/yv73vir.

The core techniques used to assess muscle fibrosis are largely consistent across experimental models (Figure 5). Time of muscle harvest varies widely across studies, with no uniformity in specific days selected. However, there were trends across studies with early timepoints (1–7 days) used to evaluate inflammatory responses, intermediate (7–14 days) to investigate regeneration and fibrosis initiation, and late (28 days and longer) to investigate sustained fibrosis. This largely replicates the same timeline seen in human muscle fibrosis [100]. Hematoxylin and eosin (H&E) staining was used to evaluate overall tissue morphology and injury progression, while picrosirius red and Masson’s trichrome staining specifically identify collagen deposition and enable quantification of fibrotic burden. Immunohistochemistry further characterizes the molecular composition of fibrotic tissue, with commonly used markers including collagen I, fibronectin, and profibrotic signaling molecules [32].

Fluorescence-activated cell sorting (FACS) is frequently employed to identify and characterize cell populations that drive fibrosis, including fibroblasts and inflammatory cell subsets [32,50,54,64]. FACS is also commonly used to study satellite cells responding to the injury, in particular to quantify them or isolate them for transcriptomic analysis [50,85,101]. More recently, transcriptomic profiling approaches, which have been predominantly applied in volumetric muscle loss (VML) models, have enabled deeper interrogation of the cellular and molecular landscape of muscle injury. Bulk RNA sequencing has been used to assess the transcriptome of whole muscle tissue following injury or intervention. This tool can specifically be used to compare how overall gene expression in the muscle changes under different conditions such as aging or injury [101].

Single-cell RNA sequencing (scRNA-seq) has revealed cell-type-specific changes and mechanisms underlying fibrosis and regeneration [24,67]. Compared to bulk sequencing, scRNA-seq provides the distinct advantage of increasing resolution of cell populations and cell-specific expression [102]. Although early studies demonstrated the efficacy of antifibrotic interventions at a phenotypic level, scRNA-seq has provided mechanistic insight into the pathways involved, the specific cell populations mediating fibrosis, and how these processes may be modulated to promote regeneration [24]. Another technique that occurs on the single-cell or single-nuclei level is the assay for transposase-accessible chromatin using sequencing (ATAC-seq). ATAC-seq is a specialized form of sequencing that requires a relatively small number of cells and can be used to understand chromosome accessibility of different genes within a cell type, along with how this might be affected by injury or other perturbation [103]. In muscle studies, single nuclei ATAC-seq has been employed to better understand the behavior of satellite cells in muscle injury [104].

Spatial transcriptomic approaches have further advanced the understanding of specific cells and mechanisms in muscle fibrosis. These techniques allow for not only gene expression information but also the context of cell populations’ spatial relationships with each other [105]. In a VML model, Larouche et al. integrated spatial transcriptomics with scRNA-seq to link molecular signatures with histopathological changes, identifying discrete fibrotic regions and migration patterns of profibrotic cells that may impede muscle stem cell infiltration and regeneration [64].

While functional testing varies across studies, it commonly includes in vivo and/or ex vivo assessment of muscle performance following injury and/or therapeutic intervention. Beyond confirming that fibrotic remodeling is associated with a corresponding loss of muscle function, functional testing is also used to evaluate the physiological and potential clinical impact of experimental interventions [24]. Measures of maximal force generation and twitch dynamics are among the most frequently reported functional outcomes. In vivo assessments typically involve neural or direct muscle stimulation to quantify twitch and maximal force production [24,32]. In contrast, ex vivo testing applies direct electrical stimulation to isolated muscles maintained in a culture bath, allowing assessment of intrinsic contractile properties independent of neural input [32,70]. Less commonly, studies incorporate gait analysis, grip testing, coordination, or endurance-based functional assays to assess whole-animal functional impairment and recovery [32,55,93].

## 4. Summary and Future Directions

While skeletal muscle can undergo near-complete regeneration following minor injury, severe injury frequently leads to fibrosis, resulting in functional impairment and an increased risk of re-injury. Significant muscle injury occurs across diverse clinical contexts, including traumatic accidents, surgical excisions, and sports-related injuries [21,106]. Much of the existing literature has focused on satellite cells and regeneration, whereas comparatively less attention has been directed toward fibrosis itself. Current experimental models of muscle injury therefore provide important opportunities to further interrogate fibrotic processes and identify strategies to prevent long-term dysfunction.

Myotoxin injection models are highly reproducible and induce uniform injury across an entire muscle. However, they lack direct clinical relevance and typically result in only transient fibrosis, limiting their utility for studying fibrotic mechanisms [50]. In contrast, laceration models offer greater pathophysiological relevance and reliably generate fibrosis [37]. However, these models may underestimate fibrosis levels seen clinically, and overlap with VML models when re-approximation is incomplete, underscoring the need for consistent terminology and detailed reporting of surgical methodology.

VML models produce the most robust and reproducible fibrosis and are well suited for studying severe muscle trauma or surgical resections in which substantial tissue is removed. Consequently, much of the VML literature emphasizes biomaterial and scaffold-based strategies to restore structure and promote regeneration. Additionally, the large fibrotic burden enables in-depth cellular and molecular analyses [64,107]. However, the sharply defined excisions characteristic of VML models may not fully capture the complex mechanical forces and heterogeneous damage seen in high-energy traumatic injuries.

Overall, existing experimental models provide valuable platforms for advancing our understanding of fibrotic mechanisms in skeletal muscle scarring. Laceration and VML models, in particular, offer consistent and reliable induction of fibrosis. Continued refinement of these models will be necessary to more accurately recapitulate the clinical pathways leading to muscle fibrosis and long-term functional impairment. In addition, detailed descriptions of surgical procedures are needed to ensure replicability between studies, and to ensure ability to isolate mechanisms involved in inducing fibrosis. More standardization among injury protocols would also be useful as many papers use varying muscle locations (for example TA versus quadriceps), defect sizes or myotoxin doses, and time points for evaluation.

Beyond the limitations of individual mouse model types, an overarching challenge across these models is the lack of clinically available therapies to reduce muscle fibrosis. Mouse and rat studies comprise the majority of existing work and have identified numerous targets with translational potential. However, future work should include studies in larger animals, which more closely replicate the human pathophysiology of muscle scarring, such as sheep, dogs, or rabbits [108]. Large animal models offer important translational advantages for understanding and managing human muscle injury and fibrosis. Volumetric muscle loss (VML) models have been developed in ovine, canine, and porcine species and provide defect sizes and injury biomechanics that more closely resemble those seen in humans. One canine VML study performed integrated transcriptomic, proteomic, and morphologic analyses to compare temporal and regional wound-healing responses with human VML injury, demonstrating translational relevance for studying human muscle fibrosis [109]. Despite this promise, canine VML models remain rare and have mainly been used to test bioengineering approaches such as scaffold-based therapies [110]. Ovine models have been more frequently used in large animal VML research, largely to evaluate tissue engineering and regenerative strategies for muscle repair [111,112,113]. Across large animal studies, however, the primary emphasis has been on testing bioengineering interventions rather than directly interrogating the mechanisms and progression of fibrosis. Only one identified study specifically focused on fibrosis in a large animal VML setting, using a porcine model to examine fibrotic remodeling and pharmacologic mitigation following injury [114]. Non-VML injury paradigms are also underrepresented in large animals. Myotoxin-based injury models have been rarely employed, with only a single study reporting the use of bupivacaine-induced muscle injury in an equine model to study regeneration [115]. To our knowledge, laceration-based skeletal muscle injury models have not been investigated in large animal systems. Given the improved similarity of large animals to humans in anatomy, biomechanics, and fibrotic and immune responses, future studies should consider using large animal models to specifically study muscle fibrosis.

Continued exploration of therapeutic interventions for muscle fibrosis is also an important area of future research. For example, recent studies have elucidated that angiotensin receptor blockers (ARBs) indirectly inhibit TGF-β1 production and therefore reduce fibrosis (Figure 2) [17,116]. This antifibrotic agent has been used in mouse models of muscle fibrosis, but little is known about its impact on human muscle fibrosis. Other examples are the use of stem cell-based therapies and platelet-enriched plasma (PRP). Stem cell-based therapies typically involve the injection of multipotent cells such as bone marrow mesenchymal stem cells (BMSCs), adipose-derived stem cells (ADSCs), and others [117]. PRP is a method by which a patient’s own blood is centrifuged to separate the plasma and the individual cell types in the blood. Platelets and other growth factors are then injected back into the patient [18]. While both methods aim to improve formation of new muscle tissue and reduce fibrosis by reducing the level of inflammatory cytokines, they each face limitations to fully achieving this goal. Stem cell-based therapies have the risk of possible rejection or immunogenic response [117]. PRP is limited by the variability in the preparation and application among clinical studies and the lack of overwhelming evidence of a clear benefit [18]. Still, both have presented promising approaches to improving muscle healing in both preclinical [118,119,120,121] and early clinical [122,123] studies. Current experimental treatment approaches, including supplementation of growth factors, gene therapy, stem cell therapy, and PRP, have largely been conducted in mice and would additionally benefit from validation in larger animal models [16,116].

Another area for future work and eventual clinical application could be finding ways to implement regeneration-promoting biomaterials to other muscle injury models aside from VML. While VML has the most obvious application for biomaterials due to a physical defect, the ability to implement satellite cells, growth factors, or other therapeutics through a hydrogel or other material could greatly benefit the field of muscle fibrosis more broadly.

## 5. Conclusions

There remains a critical need for continued investigation into the pathways and mechanisms that drive muscle fibrosis. Existing outcome measures enable reliable identification and quantification of fibrosis following skeletal muscle injury, while emerging approaches provide opportunities to interrogate the specific molecular and cellular processes that regulate fibrotic remodeling. To study these mechanisms effectively, experimental models must reliably capture clinically relevant pathophysiological features of fibrosis development. Based on the current literature, we recommend the use of laceration and volumetric muscle loss (VML) models to study the contribution of fibrosis to muscle injury. We further encourage continued refinement of these models and expansion to larger animals to enhance their pathophysiological fidelity and translational relevance to human muscle scarring.

## Figures and Tables

**Table 1:** Summary of the most common mouse skeletal muscle injury models.

Common Muscles	Technique Summary	References
**VML**		
Quad, TA	Biopsy punch ablation: A sterile circular punch tool (ranging from 1 mm to 5 mm in diameter) pressed into the muscle to remove a full or partial thickness defect.	Castor-Macias et al., 2023 [24]; Wang et al., 2019 [25]; Jacobsen et al., 2023 [26]; Rogers et al., 2021 [27]; Corona et al., 2017 [28]
	Surgical resection: A scalpel or surgical scissors used to excise a pre-defined rectangular segment or a specific weight of muscle tissue from the target area	Nuutila et al., 2017 [29]; Matthias et al., 2018 [30]; Sicari et al., 2012 [31]; Quarta et al., 2017 [32]; Quarta et al., 2018 [33]
	Multi-biopsy punch ablation: Two or more adjoining or overlapping biopsy punches made in sequence to expand the size of the ablation.	Hu et al., 2022 [34]
**Laceration**		
TA, Gastrocnemius	Partial: Transverse incision through 60% of muscle length, through 75% of width and 50% of thickness.	Menetrey et al., 1999 [35]; Hara et al., 2018 [36]
	Full-Thickness: Transverse incision through 50% of the muscle width and 100% of thickness at the widest point.	Fukushima et al., 2001 [37]; Shen et al., 2005 [38]; Zhu et al., 2007 [39]; Murray et al., 2017 [40]
	Lateral Full-Thickness: Transverse full-thickness laceration of the lateral gastrocnemius at its widest point, extending from the central neurovascular complex to the lateral muscle edge (~4 mm).	Corbiere et al., 2017 [41]
**Myotoxin Injection**		
TA, Gastrocnemius, EDL	CTX injection: Injection of 50–100 µL of 10 µM, or 20 µL of 70µM CTX.	Song et al., 2024 [42]; Guardiola et al., 2017 [43]; Feng et al., 2023 [44]; Yao, Y. et al., 2025 [45]
	NTX injection: Injection of 10 µL of 10 or 25 µg/mL NTX, often with an insulin syringe.	Brigitte et al., 2010 [46]; Tierney and Sacco, 2016 [47]; Yao, L. et al., 2021 [48]
	BaCl_2_ injection: Injection of either 10 or 50 µL of 1.2% BaCl_2_ solution.	Morton et al., 2019 [49]; Hardy et al., 2016 [50]; Li et al., 2020 [51]; Tierney and Sacco, 2016 [47]
	Re-injury model: Injection of CTX, NTX, or BaCl_2_ and repeated injury 28 days later	Hardy et al., 2016 [50]

Abbreviations: VML, volumetric muscle loss; Quad, quadriceps; TA, tibialis anterior; EDL, extensor digitorum longus; CTX, cardiotoxin; NTX, notexin; BaCl_2_, barium chloride. Note that in CTX and NTX injection models, there is a lack of consistent volumes and concentrations for injections in the literature, so we have listed the most representative protocols.

## Data Availability

No new data were created or analyzed in this study.

## Appendix A

All searches were conducted with PubMed and focused on papers published in the last 10 years. The search structure of this review first involved conducting an overall search for mouse models of muscle fibrosis using the search terms “skeletal muscle”, “injury”, “fibrosis”, and “mouse model”. After determining the most common types of mouse models, separate literature searches were conducted for the most common types: VML, laceration, and myotoxin injection. Search terms are also included for other models and large animal models.

VML: PubMed search terms included “volumetric muscle loss”, “mouse”, “skeletal muscle”, “mouse model”, “fibrosis”, and “muscle repair”.

Laceration: PubMed search terms included “skeletal muscle”, “laceration”, “fibrosis” “mouse”, “muscle”, and “muscle fibrosis”.

Myotoxin: PubMed search terms included “skeletal muscle”, “toxin injection”, “mouse model”, “cardiotoxin”, “notexin”, “barium chloride”, “bupivacaine”, “local anesthetic”, “injection”, and “mouse”.

Other Models: PubMed searches were conducted with the terms “ischemia/reperfusion”, “ischemia”, “mouse model”, “skeletal muscle”, and “muscular dystrophy”.

Large animal models: PubMed search terms included “skeletal muscle”, “sheep”, “dog”, “cat”, “horse”, “large animal”, “volumetric muscle loss”, “laceration”, “fibrosis”, “muscle repair”, “barium chloride”, “bupivacaine”, “cardiotoxin”, “notexin”, AMD “myotoxin”.
